# A RT-FDTD method of analyzing wireless propagation characteristics in underground mine

**DOI:** 10.1038/s41598-024-60151-1

**Published:** 2024-04-29

**Authors:** Xiaoyan Song, Gaomin Zhang, Chang Zhou

**Affiliations:** 1 College of Electrical and Mechanical Engineering, Pingdingshan University, Pingdingshan, 467000 China; 2 College of Information engineering, Pingdingshan University, Pingdingshan, 467000 China; 3 Information Technology Research Institute, China University of Mining and Technology-Beijing, Beijing, 100083 China; 4 School of Computer Science and Technology, Henan Open University, Zhengzhou, 450008 China

**Keywords:** Mine, Wireless propagation characteristics, Ray tracing, Finite difference time domain, Simulation efficiency, Computational science, Computer science, Information technology

## Abstract

Efficient communication is crucial in reducing injuries and fatalities in coal mine accidents, necessitating the study of simulation methods for mine communication. When transceiver antennas are positioned close to the same side of the tunnel, the simulation results from the Ray Tracing (RT) method exhibit significant errors. Additionally, the Finite-Difference Time-Domain (FDTD) method demands substantial computational resources. In response to these challenges, we propose a RT-FDTD method, guided by the law of conservation of energy. This approach involves dividing the mine tunnel into a cuboidal region, using the RT method to calculate the electric field strength on the cuboid’s surface, and then employing this as the excitation source for the FDTD method. Subsequently, the FDTD method is used to calculate the electric field strength within the cuboid. Experimental results demonstrate that the RT-FDTD method effectively mitigates the limitations of the RT and FDTD methods, enhancing both the efficiency and accuracy of simulations in underground mine.

## Introduction

Wireless communication systems play an important role in the intelligent, unmanned, safe production and emergency rescue of coal mine^1^. The wireless propagation environment in underground mine is quite different from that on the ground due to the complex geometry and material properties of the mine tunnel^2^. To prevent disruption to pedestrian or vehicle traffic, wireless communication base station antennas in mines are installed near the tunnel wall or roof. Numerous methods and measurement experiments have been conducted to study wireless propagation characteristics in underground mine. However, many of these studies focus solely on the transmitting and receiving antennas located in the center of the tunnel section. Given the planning requirements for the antenna position of the mine wireless communication system, it is essential to study the wireless propagation characteristics when the receiving and transmitting points are near the tunnel wall or roof, not just the central position.

Several wireless propagation models have been proposed for mine environments, such as waveguide mode models^3^, ray tracing (RT)^4^, finite-difference time domain (FDTD)^5,6^, parabolic formula (PE)^7^, two slope loss^8^, and hybrid computing models^9^. However, these models have some limitations in simulating the electromagnetic waves in mine. The waveguide mode model is further divided into single waveguide mode model and multi waveguide mode model, and the single wave mode model is only applicable to the calculation of propagation characteristics in the far-field region of the fundamental mode^10^, and cannot calculate the propagation characteristics of high-frequency electromagnetic waves in large cross-section and long-distance tunnels in mine^11^. The multi waveguide mode model is suitable for calculating the wireless propagation characteristics of mine in the near and far regions at multiple frequencies, but cannot calculate the impact of complex environments such as tunnel branches or tunnel wall roughness on the wireless propagation characteristics of mine^12^. The RT method treats electromagnetic waves as light rays. When the environmental size is greater than the electromagnetic wave length, accurate results can be obtained^13^, but RT method cannot calculate the wireless propagation characteristics when the transmitting and receiving antennas are simultaneously close to the same side of the tunnel^14^. The FDTD method is a comprehensive wave analysis technique. It can calculate the wireless propagation characteristics in complex mine environments with high accuracy. However, the FDTD method requires substantial computational resources to simulate electromagnetic wave characteristics in underground mine. This demand for computing space and power can be a significant limitation in its application^15^.

Critical factors for a method analyzing wireless propagation characteristics include required computer memory space, simulation time, and accuracy. These factors are particularly significant for the FDTD method, as they are closely related to the tunnel volume and electromagnetic wave frequency^16^. Therefore, the FDTD method faces challenges in calculating the propagation characteristics of high-frequency electromagnetic waves in tunnels with large cross-sections and long distances. Several methods have been proposed to overcome the limitations of the Courant Friedrichs Lewy (CFL) condition. These include the Alternating Direction Implicit Finite-Difference Time-Domain (ADI-FDTD) method^17^, the Locally 1-D FDTD method (LOD-FDTD)^18^, and the Split-Step and Crank-Nicolson (SSCN-FDTD) method^19^. However, these methods tend to increase the numerical dispersion error when the time step is larger. Furthermore, these methods are typically used for simulations in small spaces due to their computational constraints. The methods for solving parabolic equations are Split Step Fourier Transform (SSFT) and finite difference (FD), where SSFT is not easy to deal with irregular boundaries. When using FD to solve parabolic equations, the iteration step size is strictly limited by the electromagnetic wave length, which requires allocating memory for a large number of matrices, making it difficult to solve the propagation characteristics of high-frequency electromagnetic waves in large-scale space^20^. The dual slope loss empirical model is derived from the dual ray theory and is suitable for studying the propagation characteristics of mine electromagnetic waves when both the transmitting and receiving antennas leave the tunnel for several wavelengths and the transmitting and receiving antennas are visible^8^.

In this paper, we propose a RT-FDTD method that combines RT and FDTD to analyze the wireless propagation characteristics in underground mine. The proposed method divides the mine tunnel into a cuboid area and uses RT to calculate the electric field strength on the surface of the cuboid as the excitation source of FDTD. Then, FDTD is used to calculate the electric field strength inside the cuboid. Each model operates within its own optimal scenario, leveraging the unique advantages of different computing methods. The experimental results demonstrate that the RT-FDTD method can effectively mitigate the limitations of both the RT and FDTD methods, thereby enhancing the efficiency and accuracy of simulations in underground mine tunnels. This method provides a valuable tool for the design and optimization of wireless communication systems in underground mine. Our main contributions can be summarized as follows:

- We propose a RT-FDTD method to analyze the propagation characteristic analysis in underground mine, which combines RT method’s high efficiency and FDTD method’s precision using law of conservation of energy; and it expands application range for RT method and FDTD method in wireless propagation characteristics analysis for a mine.
- The RT-FDTD method will calculate signal strength for different frequency electromagnetic waves consistently with results calculated by FDTD method and unit window recycling FDTD (UWR-FDTD) method as well as measured values in this paper, no matter where the transmitting and receiving antennas are located in the tunnel section.
- The RT-FDTD method greatly improves the efficiency of electromagnetic wave propagation characteristics in technical mine and has high computational accuracy.

The rest of this paper is organized as follows. In “RT-FDTD model” section, we introduce the RT-FDTD model. In “Numerical calculation and analysis” section, we conduct simulation experiments and underground measurement experiments using this model. Following this, we analyze the accuracy and efficiency of the simulation data. The paper concludes in “Conclusions” section.

## RT-FDTD model

### RT-FDTD principle

Combining RT and FDTD method has been used to improve LED correlated color temperature uniformity^21^ and set multiscale optical simulation^22^,and the needed computational space is relatively small in these application scenarios. The RT method is one of the main analytical methods for simulating the characteristics of wireless propagation in long and straight tunnels^23^. It treats the high-frequency electromagnetic waves radiated from the transmitter as multiple rays, and expresses the electric field strength at a point in the tunnel by the sum of the direct and reflected rays arriving at that point. The RT method can accurately calculate the electromagnetic wave properties in the tunnel as long as the transmitter and receiver are not close to the same side of the tunnel wall at the same time. FDTD method calculation of wireless propagation characteristics in mine requires discretizing the whole tunnel space into multiple Yee grids. Based on the spatial and temporal relationships between the electric and magnetic fields, the electric and magnetic field strengths can be calculated for each Yee grid. The FDTD method has a high computational accuracy throughout the tunnel. In order to prevent numerical dispersion of electromagnetic waves during propagation after spatial discretization^24^, the spatial step of the Yee grid generally does not exceed 1/10 of the electromagnetic wave wavelength^25^, while the time step and spatial step also need to satisfy CFL condition^26,27^. Restricted by these two conditions, the higher the electromagnetic wave frequency, the shorter the Yee grid spatial step, and the more Yee grids need to be divided within the same tunnel.Indeed, while some methods^17– 19^ have made strides in addressing this limitation, it’s important to note that there is a close relationship between computational error and spatial step size. This relationship can significantly impact the accuracy of simulations, making it a crucial factor to consider in the development and application of these methods. The direct use of the FDTD method in the tunnel to calculate the wireless propagation characteristics requires huge memory space and long simulation time.

**Figure 1:**
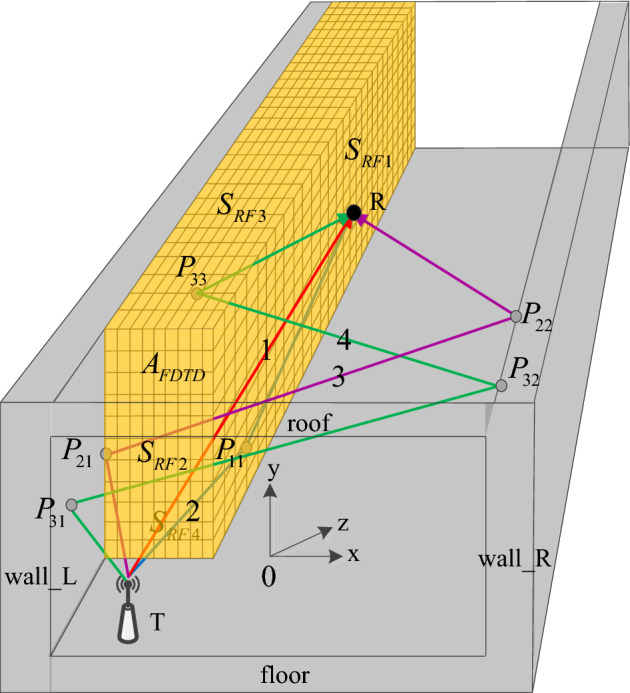
Schematic of the RT-FDTD model.

In the RT-FDTD model, a vertical surface is set along the length of the tunnel from an area near the center of the tunnel cross-section, and this vertical surface forms a cuboid region with the tunnel roof, one side of the tunnel wall, and the tunnel floor. The vertical surface can also form a cuboid area with a plane parallel to the tunnel roof, a side of the tunnel, and a plane parallel to the tunnel floor. The cuboid region is divided into two parts: the surface of the cuboid body and the internal space of the cuboid body. The size of the grid in the internal space of the cuboid and the size of the discrete grid on the surface of the cuboid are determined by the FDTD method. The electric field strength of each discrete grid on the surface of the cuboid is calculated by RT method. The inside region of the cuboid is discretized with Yee grids, and the FDTD iterative formula is executed to calculate the electromagnetic field strength in the cuboid. According to the law of conservation of energy, the electric field strength of the entire surface of the cuboid obtained by the RT method can be used as the excitation source of the FDTD method. The RT-FDTD method can greatly reduce the memory space and simulation time required by FDTD method in the same simulation space, and ensure the calculation accuracy and calculation efficiency when the transceiver point is located near the tunnel wall at the same time. The schematic for the RT-FDTD model is shown in Fig. 1. The x direction is the tunnel width direction, the y is the tunnel height direction, and the z is the tunnel length direction.

In Fig. 1, the gray areas of the tunnel roof, floor and both sides of the tunnel are the tunnel walls. These four surfaces form a complete simulation of the tunnel using the same electromagnetic parameters. To give a clearer picture of Fig. 1, the tunnel roof is not represented in gray, only a discrete cuboid region named $A_{FDTD}$ is delineated for the area near wall L, which is discretized with a Yee grid. The calculation area for the RT method is the entire tunnel space, and the emission point T can be located anywhere within the tunnel section. The surfaces labeled $S_{RF1}, S_{RF2}, S_{RF3}$ and $S_{RF4}$ are the interfaces of the calculation areas of RT and FDTD. $S_{RF1}$ is close to and parallel to the tunnel wall L, $S_{RF2}$ is close to the emission point and parallel to the coordinate plane *xoy*, $S_{RF3}$ is close to and parallel to the roof, and $S_{RF4}$ is close to and parallel to the floor. Assuming that the receiving point R is on the face $S_{RF1}$, we calculate the electric field strength at the receiving point R using the RT method, with the ray labelled 1 indicating the direct path. The ray labelled 2 represents the first reflection path after being reflected by the floor, and the first reflection point is $P_{11}$. The ray labeled 3 represents the second reflection path after being reflected by the wall L and R, and the second reflection points are $P_{21}$ and $P_{22}$, respectively. The ray 4 represents the 3rd reflection path after being reflected by the wall L, R and L successively, and with the 3rd reflection points are $P_{31}$, $P_{32}$ and $P_{33}$ respectively, where the reflection points $P_{31}$ and $P_{33}$ are located on the wall L and the reflection point $P_{32}$ is located on the wall R. The yellow grid region of the cuboid $A_{FDTD}$ is the FDTD method computing space, and the size of each Yee grid in this region determine the spatial resolution of the RT method to calculate the electric field strengths on the surfaces named $S_{RF1}, S_{RF2}, S_{RF3}$ and $S_{RF4}$. Based on the law of conservation of energy, the electric field strengths on surfaces named $S_{RF1}, S_{RF2}, S_{RF3}$ and $S_{RF4}$ can be used as the excitation source for calculating the interior region of the $A_{FDTD}$ using the FDTD method and calculate the electric field strengths for each Yee grid by the standard FDTD iterative formulas.

Let the coordinate origin be at the center of the tunnel section, the tunnel width is W, the tunnel height is H and the tunnel length is L also the coordinate range of surface $S_{RF1}$ along the width (*x* direction), height (*y* direction) and length (*z* direction) of tunnel be satisfied

1 $$\begin{aligned} {\left\{ \begin{array}{ll} -\frac{1}{4} W\le x_{RF1} \le \frac{1}{4}W \\ -\frac{1}{2}H\le y_{RF1} \le \frac{1}{2}H \\ 1\le z_{RF1} \le L \end{array}\right. } \end{aligned}$$

where $x_{RF1}, y_{RF1}, z_{RF1}$ are the coordinate values of each Yee grid in the surface $S_{RF1}$. The coordinate range of the surface $S_{RF2}$ along the width (*x*-direction), height (*y*-direction) and length (*z*-direction) of the tunnel should be satisfied

2 $$\begin{aligned} {\left\{ \begin{array}{ll} -\frac{1}{2} W\le x_{RF2} \le x_{RF1} \\ -\frac{1}{2}H\le y_{RF2} \le \frac{1}{2}H \\ 1\le z_{RF2} \le L \end{array}\right. } \end{aligned}$$

where $x_{RF2}, y_{RF2}, z_{RF2}$ are the coordinate values of each Yee grid in the surface $S_{RF2}$. The coordinate range of surface $S_{RF3}$ along the width (*x*-direction), height (*y*-direction) and length (*z*-direction) of the tunnel be satisfied

3 $$\begin{aligned} {\left\{ \begin{array}{ll} -\frac{1}{2} W\le x_{RF3} \le x_{RF1} \\ 0 < y_{RF3} \le \frac{1}{2}H \\ 1\le z_{RF3} \le L \end{array}\right. } \end{aligned}$$

where $x_{RF3}, y_{RF3}, z_{RF3}$ are the coordinate values of each Yee grid in the surface $S_{RF3}$. The coordinate range of surface $S_{RF4}$ along the width (*x*-direction), height (*y*-direction) and length (*z*-direction) of the tunnel be satisfied

4 $$\begin{aligned} {\left\{ \begin{array}{ll} -\frac{1}{2} W\le x_{RF4} \le x_{RF1} \\ -\frac{1}{2} H \le y_{RF4} < 0 \\ 1\le z_{RF4} \le L \end{array}\right. } \end{aligned}$$

where $x_{RF4}, y_{RF4}, z_{RF4}$ are the coordinate values of each Yee grid in the surface $S_{RF4}$.

### Calculation of the RT region

**Figure 2:**
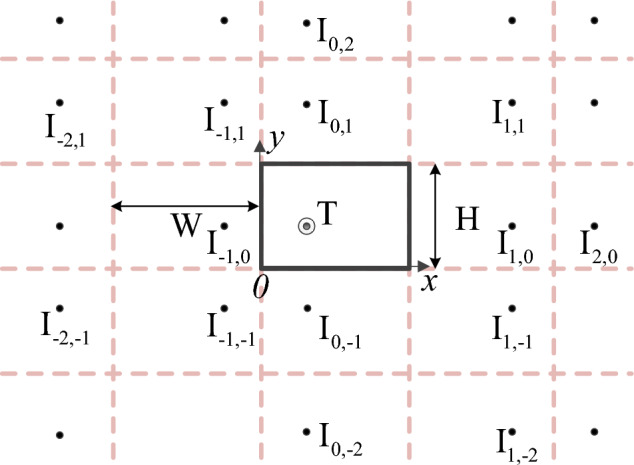
Different image points of the emitter T.

According to (1), the distance between the surface $S_{RF1}$ and the tunnel wall along the coordinates in the *x* direction is at least 1/4 of the tunnel width. In this case, the distance between the transmitting point and the tunnel wall does not affect the accuracy of calculating the electric field strength on the surface $S_{RF1}$ by the RT method, which avoids the shortcoming of large error when the RT method is used to calculate the transceiver antenna at the same time near the tunnel wall. Set the origin of coordinates at the center of the tunnel, and set the coordinate of the transmitting point T as $(x_0, y_0, 0)$, then the image point coordinates of the transmitting point with respect to the four tunnel walls are satisfied

5 $$\begin{aligned}  x_{p} =W_{p}+\left( -1 \right) ^{p} x_{0}\\ y_{q} =H_{q}+\left( -1 \right) ^{q} y_{0} \end{aligned}$$

where *p* and *q* are integers. A positive *p* indicates the number of reflections of the transmitting point relative to the tunnel roof and a negative *p* indicates the number of reflections from the tunnel floor. A positive *q* indicates the number of reflections from the transmitting point relative to the tunnel wall with positive coordinates and a negative *q* indicates the number of reflections from the transmitting point relative to the tunnel wall with negative coordinates. Figure 2 shows a schematic diagram of solving for the partial image points of the transmitting point T on different wall surfaces. The width of the simulated tunnel is 2*a* and the height is 2*b*.

The distance between the ray radiated by the transmitting point T and the receiving point R after reflection *p* and *q* times is equal to the distance between the image point I_p,q_ of the transmitting point and the receiving point R. Assuming that the coordinate of the receiving point R is (*x*, *y*, *z*), then the distance named $r_{pq}$ from R to the image point I_p,q_ is satisfied

6 $$\begin{aligned} r_{p,q}=\sqrt{\left( x-x_{p} \right) ^{2} + \left( y-y_{q} \right) ^{2}+z^{2} } \end{aligned}$$

According to RT theory, the Angle between incident ray and normal vector of tunnel wall, roof or floor is set as $\theta _{\parallel }$ and $\theta _{\perp }$ respectively, and the magnitude satisfies can be expressed as

7 $$\begin{aligned} \theta _{\parallel } = \arccos \left\{ \frac{\left| x-x_{p}\right| }{r_{p,q} } \right\} \\ \theta _{\perp } = \arccos \left\{ \frac{\left| y-y_{q}\right| }{r_{p,q} } \right\} \end{aligned} $$

The incident wave electric field vector can be decomposed into horizontal and vertical polarized electric field vectors, named $E_{t\parallel }$ and $E_{t\perp }$

8 $$\begin{aligned}  E_{t\parallel }&= \left| E_{t} \right| \cos \theta _{\parallel } \frac{{\varvec{n}}\times {\varvec{r}}}{\left| {\varvec{n}}\times {\varvec{r}} \right| } \\ E_{t\perp }&= \left| E_{t} \right| \cos \left( \frac{\pi }{2} -\theta _{\perp } \right) \frac{{\varvec{n}}\times {\varvec{r}}}{\left| {\varvec{n}}\times {\varvec{r}} \right| } \end{aligned}$$

where ${\varvec{n}}$ is the normal vector of the plane where the reflection point is located, ${\varvec{r}}$ is the incident wave direction vector.

Assuming that the same building materials are used so that the electromagnetic parameters are the same on tunnel walls. The reflection coefficients on both sides of tunnel walls and roof and floor can be obtained by using the angle between incident rays and reflective surfaces and the electromagnetic parameters of the walls, named $\Gamma _{\parallel }$ and $\Gamma _{\perp }$

9 $$\begin{aligned} \Gamma _{\parallel } =\frac{\overline{\varepsilon _{r} }\cos \theta _{\parallel } -\sqrt{\overline{\varepsilon _{r} }-\sin ^{2} \theta _{\parallel } } }{\overline{\varepsilon _{r} }\cos \theta _{\parallel } +\sqrt{\overline{\varepsilon _{r} }-\sin ^{2} \theta _{\parallel } } } \\ \Gamma _{\perp } =\frac{\cos \theta _{\perp } -\sqrt{\overline{\varepsilon _{r}}-\sin ^{2}\theta _{\perp }}}{\cos \theta _{\perp } +\sqrt{\overline{\varepsilon _{r}}-\sin ^{2}\theta _{\perp }}} \end{aligned}$$

where $\overline{\varepsilon _{r} } =\varepsilon _{r}-\left( j\sigma /\omega \varepsilon _{0} \right)$ is the complex dielectric constant of tunnel wall, $\varepsilon _{r}$ is the relative dielectric constant of tunnel wall, $\omega$ is the angular frequency of electromagnetic wave, $\sigma$ is the conductivity of tunnel wall, $\varepsilon _{0}$ is the dielectric constant of vacuum.

The RT method calculates an expression for the electric field strength at the receiving point based on the length of the direct path, the length of the reflected path for different numbers of reflections, and through Eq. (8).

According to the length of the direct path and the length of the reflected path with different reflection times, the expression of the electric field strength Er at the receiving point can be calculated by Eq. (8), that is

10 $$\begin{aligned} E_{r} =\sum _{p=-\infty }^{p=\infty }\sum _{q=-\infty }^{q=\infty } \left[ \frac{exp\left( -jkr_{p,q} \right) }{r_{p,q}} \right] \left( E_{t\perp }\Gamma _{\perp }^{\left| p \right| }+ E_{t\parallel }\Gamma _{\parallel }^{\left| q \right| } \right) \end{aligned}$$

where j is an imaginary number unit, k is the electromagnetic wave number. As shown in Fig. 1, the electric field strengths on $S_{RF1}, S_{RF2}, S_{RF3}$ and $S_{RF4}$ are calculated using Eq. (10) as the excitation source for the FDTD calculation region inside the cuboid $A_{FDTD}$, respectively. The spatial sampling intervals for the electric fields on each of the above surfaces obtained by the RT method are the same as the Yee grid sizes of the FDTD calculation region.

### RT-FDTD calculation region

**Figure 3:**
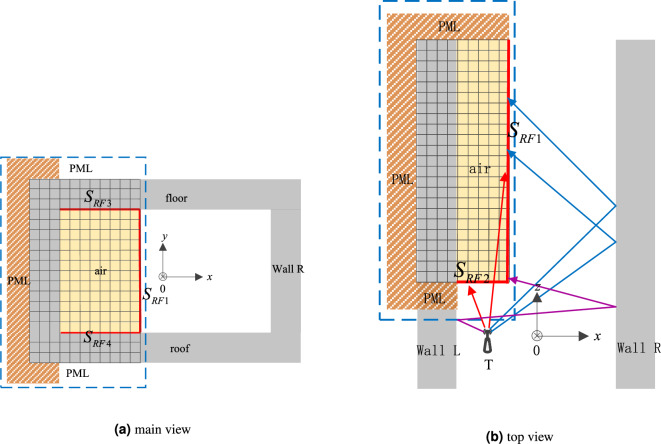
The region model calculated by RT-FDTD.

Modeling the entire tunnel using FDTD method directly requires huge memory space. Firstly, the RT method is used to calculate the electric field on a surface near the tunnel center, which is used as the excitation source of the FDTD calculation area. Then, the FDTD method is used to model the area near the tunnel wall. The RT-FDTD method avoids the direct division of Yee grid in the entire tunnel space, saves a lot of memory space and simulation time, and improves simulation efficiency. Figure 3 shows the model for calculating the internal spatial electric field strength of the cuboid $A_{FDTD}$ in Fig. 1 using the FDTD method.

The area surrounded by blue dashed lines in Fig. 3 is the FDTD calculation region, which mainly includes three parts, that is, Perfectly Matched Layer (PML), including tunnel space, excitation source surface. The tunnel space is filled with air medium, and the electric fields on $S_{RF1}, S_{RF2}, S_{RF3}$ and $S_{RF4}$ excitation sources composed of multiple receiving points have been calculated by RT method. PML is wrapped in the outer sides of the tunnel wall L, roof and floor to absorb the electromagnetic wave transmitted to the tunnel wall and prevent it from reflecting back to the tunnel space. The excitation source surface is directly used as a boundary of the FDTD calculation region, and its electric field strength is only related to the calculation results of the RT method, so it is not necessary to set the PML in the excitation source surface.

The red lines $S_{RF1}, S_{RF2}, S_{RF3}$ and $S_{RF4}$ in the main view of FDTD calculation area represent the excitation sources calculated by RT method as FDTD update equation. In the top view, it can be seen that there are two surfaces, and, as FDTD excitation sources, and represents the electric field parallel to the *xoy* plane as FDTD excitation sources in the *xoy* direction. Once the *x* coordinate of surface A is determined, the number of grids that FDTD needs to divide in that direction can be calculated according to the space step size in the x direction of Yee grid. The number of Yee grids required in the y direction is determined by the *y* coordinates of surface, and the spatial step size of the Yee grids in that direction. The required number of Yee grids in the z direction is determined by the length of the simulated tunnel the difference of the length from the transmitting point to the surface and the spatial step size of the Yee grid in that direction.

The spatial step and temporal step in the FDTD iterative equations need to satisfy the CFL stability condition.

11 $$\begin{aligned} \bigtriangleup t< \frac{\sqrt{\mu \varepsilon } }{\sqrt{\left( \bigtriangleup x \right) ^{-2} +\left( \bigtriangleup y \right) ^{-2}+\left( \bigtriangleup z \right) ^{-2}}} \end{aligned}$$

Where $\bigtriangleup t$ is the discrete time step, $\bigtriangleup x, \bigtriangleup y, \bigtriangleup z$ correspond to the Yee grid spatial step size in the three directions of the coordinate axis respectively; $\mu$ is the permeability of electromagnetic wave propagation medium; $\varepsilon$ is the dielectric constant of electromagnetic wave propagation medium.

The four surface excitation sources, such as$S_{RF1}, S_{RF2}, S_{RF3}$ and $S_{RF4}$ are directly coupled to the Yee grid inside the cuboid, and iterated along the *x* direction from the surface to the wall L in accordance with the temporal step. Finally, the electric field strength distribution in the whole tunnel is obtained.

The FDTD iterative equation for solving the electric field of the Yee grid inside the cuboid are

12 $$\begin{aligned} E_{x}^{n+1} &=C_{a}E_{x}^{n}+C_{by}\left[ H_{z}^{n+\frac{1}{2} }\left( i+\frac{1}{2},j+\frac{1}{2},k\right) -H_{z}^{n+\frac{1}{2} }\left( i+\frac{1}{2},j-\frac{1}{2},k\right) \right] \\ & \quad + C_{bz}\left[ H_{y}^{n+\frac{1}{2}}\left( i+\frac{1}{2},j,k-\frac{1}{2} \right) - H_{z}^{n+\frac{1}{2} }\left( i+\frac{1}{2},j,k+\frac{1}{2}\right) \right] \end{aligned}$$

13 $$\begin{aligned} E_{y}^{n+1} &=C_{a}E_{y}^{n}+C_{bz}\left[ H_{x}^{n+\frac{1}{2} }\left( i,j+\frac{1}{2},k+\frac{1}{2}\right) -H_{x}^{n+\frac{1}{2} }\left( i,j+\frac{1}{2},k-\frac{1}{2}\right) \right] \\ & \quad + C_{bx}\left[ H_{z}^{n+\frac{1}{2}}\left( i-\frac{1}{2},j+\frac{1}{2},k \right) - H_{z}^{n+\frac{1}{2} }\left( i+\frac{1}{2},j+\frac{1}{2},k\right) \right] \end{aligned}$$

14 $$\begin{aligned} E_{z}^{n+1} &=C_{a}E_{z}^{n}+C_{bx}\left[ H_{y}^{n+\frac{1}{2} }\left( i+\frac{1}{2},j,k+\frac{1}{2}\right) -H_{y}^{n+\frac{1}{2} }\left( i-\frac{1}{2},j,k-\frac{1}{2}\right) \right] \\ & \quad + C_{by}\left[ H_{x}^{n+\frac{1}{2}}\left( i,j-\frac{1}{2},k+\frac{1}{2} \right) - H_{x}^{n+\frac{1}{2} }\left( i,j+\frac{1}{2},k+\frac{1}{2}\right) \right] \end{aligned}$$

where $E_x, E_y, E_z, H_x, H_y, H_z$ represent the electric field strength and magnetic field strength in 3 directions such as *x*, *y*, *z*, respectively. The superscripts $n, n+1/2, n+1$ for the electric and magnetic field strengths denote the discrete time step of the FDTD method. The characters *i*, *j* and *k* are integers, representing the grid coordinate values of Yee grid in the *x*, *y*, *z* directions in the cuboid. The expressions of the four coefficients in the equation are $C_{a} =\frac{2\varepsilon -\sigma \Delta t}{2\varepsilon +\sigma \Delta t}$, $C_{bx} =\frac{2\Delta t}{2\varepsilon \Delta x +\sigma \Delta t \Delta x}$, $C_{by} =\frac{2\Delta t}{2\varepsilon \Delta y +\sigma \Delta t \Delta y}$, $C_{bz} =\frac{2\Delta t}{2\varepsilon \Delta z +\sigma \Delta t \Delta z}$.

The FDTD iterative equation for solving the magnetic field of the Yee grid inside the cuboid are

15 $$\begin{aligned} H_{x}^{n+\frac{1}{2}} &=H_{x}^{n-\frac{1}{2}} +C_{cy}\left[ E_{z}^{n}\left( i,j+1,k+\frac{1}{2}\right) -E_{z}^{n}\left( i,j,k+\frac{1}{2}\right) \right] \\ & \quad + C_{cz}\left[ E_{y}^{n}\left( i,j+\frac{1}{2},k \right) - E_{y}^{n}\left( i,j+\frac{1}{2},k+1\right) \right] \end{aligned}$$

16 $$\begin{aligned} H_{y}^{n+\frac{1}{2}} &=H_{y}^{n-\frac{1}{2}} +C_{cz}\left[ E_{x}^{n}\left( i+\frac{1}{2},j,k+1\right) -E_{x}^{n}\left( i+\frac{1}{2},j,k\right) \right] \\ & \quad + C_{cx}\left[ E_{z}^{n}\left( i,j,k+\frac{1}{2} \right) - E_{z}^{n}\left( i+1,j,k+\frac{1}{2}\right) \right] \end{aligned}$$

17 $$\begin{aligned} H_{z}^{n+\frac{1}{2}}& =H_{z}^{n-\frac{1}{2}}+C_{cx}\left[ E_{y}^{n}\left( i+1,j+\frac{1}{2},k\right) -E_{y}^{n}\left( i,j+\frac{1}{2},k\right) \right] \\ & \quad + C_{cy}\left[ E_{x}^{n}\left( i+\frac{1}{2},j,k \right) - E_{x}^{n}\left( i+\frac{1}{2},j+1,k\right) \right] \end{aligned}$$

## Numerical calculation and analysis

### Analysis of calculation accuracy

While there are numerous electromagnetic simulation methods available, most are typically used to analyze small targets, with only a few being applied in underground mine environments. In our study, we have referred to various articles and selected the UWR-FDTD^16^, RT^14^, and RT-FDTD methods. These methods were used to calculate and compare the wireless propagation characteristics of 740 MHz electromagnetic waves. The environment for these simulations was a rectangular tunnel with dimensions of 200 m in length, 4.8 m in width, and 3.4 m in height. This was done to analyze the calculation accuracy of the RT-FDTD method. The construction materials of both sides of the tunnel wall, roof and floor are concrete, so they have the same electromagnetic parameters around the tunnel: a relative permittivity of 8 and a conductivity of 1e−2 S/m^28^. The Yee grid divided by FDTD method in the internal region of the cuboid has a uniform spatial grid step in all three directions. When this spatial step is 1/10 of the electromagnetic wave wavelength, it is 0.04 m and the corresponding temporal step is 70 *ps* to satisfy the CFL condition according to Eq. (9).

**Figure 4:**
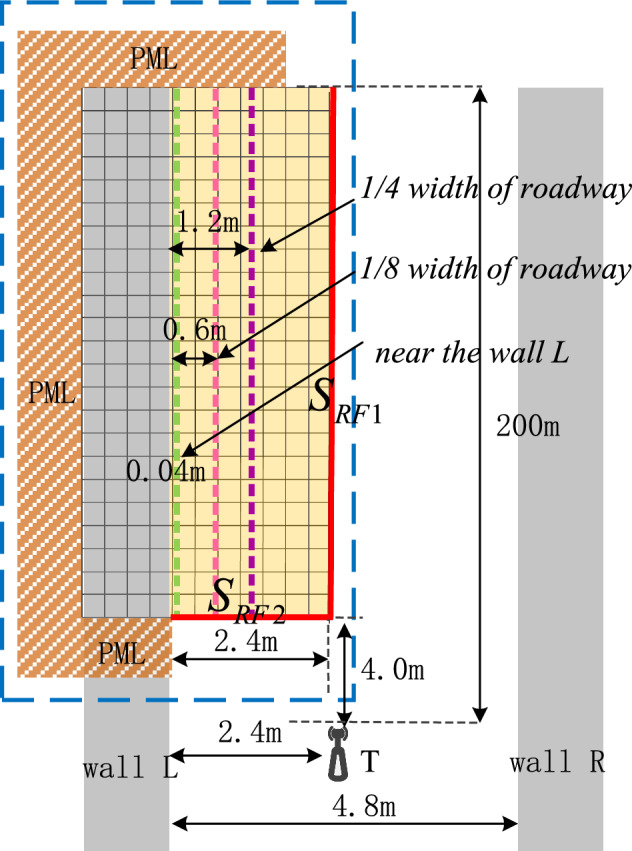
Location parameters of the transmitting point and excitation surface source.

The origin of coordinates is located in the center of the tunnel section, and we set their coordinate parameters according to the positions of the transmitting point and surface excitation source in Fig. 4.

Most numerical simulations or measurement experiments studying wireless propagation characteristics always set the transmitting and receiving points in the central area of the tunnel section. However, We all know that wireless communication base station antennas are installed on the walls or roof of tunnels. Therefore, when designing verification experiments, it is indeed crucial to not only consider the central area of the tunnel section but also the areas close to the tunnel walls. This approach will provide a more comprehensive understanding of the performance of the RT-FDTD method in real-world scenarios, which has significant practical implications.

**Figure 5:**
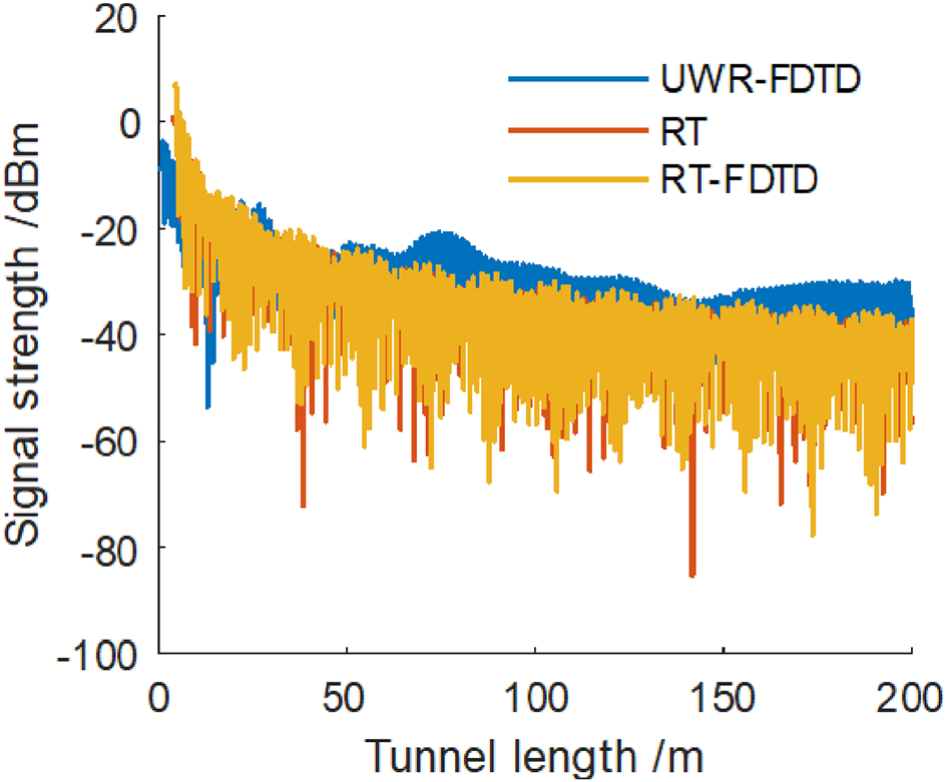
Electromagnetic wave signal strength along z direction at point (− 1.2,0).

Firstly, we calculated and compared the results when we placed the transmitting point in the center of a tunnel section with length, width and height of 200 m, 4.8 m and 3.4 m respectively; and we placed the receiving point close to a tunnel wall L at a distance of either 1/4 or 1/8 of the tunnel width. The coordinate of the transmitting point is (0,0,0). We set the x-coordinate value of a surface excitation source to be 0, which is half of the tunnel width. The distance from this surface excitation source $S_{RF2}$ to the emission point along the *z*-axis is 4 m; and the *y*-coordinates of the surface excitation source of $S_{RF2}$ and $S_{RF3}$ are 0.8 and − 0.8 respectively. We used RT method to calculate the electric field strength at 4 surface excitation sources for a 740 MHz electromagnetic wave when we placed its emission point at the center of a tunnel section. Then we used these calculated electric field strengths as FDTD excitation sources. We executed Eqs. (12–17) in a cuboid’s internal space according to required time steps along x-axis using FDTD method to solve for electric field strength at each Yee grid in a cuboid composed of surfaces $S_{RF1}$, $S_{RF3}$, $S_{RF4}$ and wall L. We used UWR-FDTD method from reference^13^ to divide eight virtual windows along *z*-axis of a tunnel with unit window length, width and height being 25 m, 4.8 m and 3.4m respectively. We placed a transceiver antenna in a tunnel with sectional coordinates (− 1.2,0), which means its distance from wall L is o1/4 of a tunnel’s width. In a same tunnel environment as UWR-FDTD’s, we used RT method from reference^16^ and our RT-FDTD method to calculate electric field strength at each receiving sampling point along *z*-axis of a tunnel. To compare with measured values, we converted calculated electric field strengths into received signal power expressed in dBm. The results are shown in Fig. 5.

**Figure 6:**
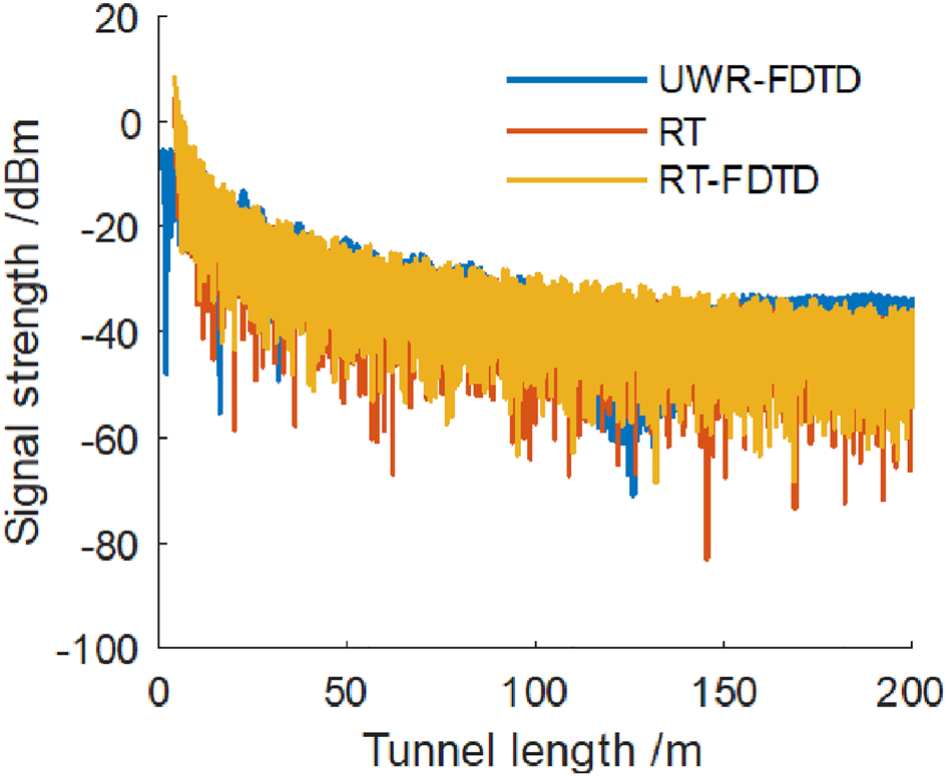
Electromagnetic wave signal strength along z direction at point (− 1.8,0).

Figure 5 shows that when we place a transmitting point at a tunnel section’s center and place a receiving point at a distance of 1/4 of a tunnel’s width from wall L, UWR-FDTD, RT and our RT-FDTD method calculate similar electromagnetic wave signal strength variation patterns for each longitudinal sampling in a tunnel. Since RT-FDTD method uses some RT method’s calculation results, RT method and our RT-FDTD method have very similar calculation results. We keep transmitting point’s position and excitation source plane’s position unchanged; and place a receiving point on a tunnel section with coordinate (− 1.8,0), which means its distance from wall L is 1/8 of a tunnel’s width as shown in Fig. 4. Figure 6 shows electromagnetic wave signal strength at each receiving sampling point calculated along z-axis of a tunnel.

In Fig. 6, when we place a transmitting point at a tunnel section’s center and place a receiving point at a distance of 1/8 of a tunnel’s width from wall L, all three calculation methods show a consistent attenuation pattern for received electromagnetic wave signal strength.

**Figure 7:**
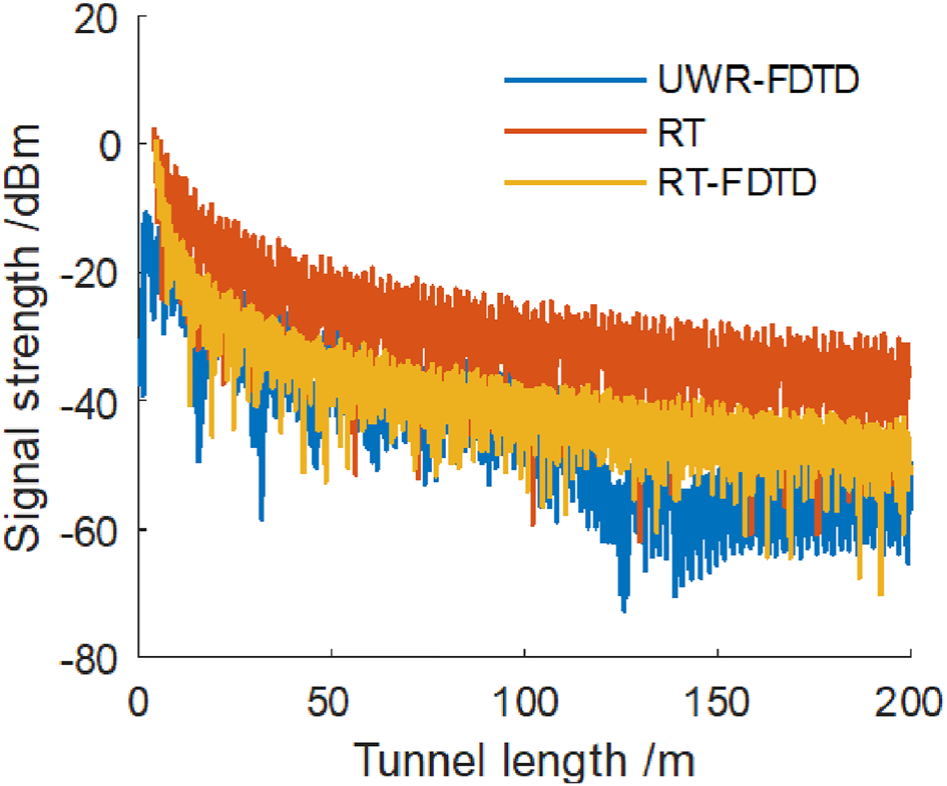
Electromagnetic wave signal strength along z direction at point (− 2.36,0).

We keep transmitting point’s position unchanged and place a receiving point at a distance of 0.04 m from a tunnel wall. When receiving sampling points are identical, RT method calculates a received signal power that is 12 dB higher than what UWR-FDTD method and our RT-FDTD method calculate; and UWR-FDTD method and our RT-FDTD method calculate identical received signal power variation patterns for each receiving sampling point. Figure 7 shows these results.

UWR-FDTD method’s iterative process resembles traditional FDTD method’s iterative process. Multipath affects signal strength calculated by this method more when it is near a tunnel wall, and large-scale fading causes more obvious amplitude fluctuations for received signal. Surface source’s distance from a tunnel wall is 2.4 m. Since RT-FDTD method’s excitation source mainly comes from RT method, RT-FDTD method calculates a relatively gentle amplitude fluctuation for received signal strength. RT method cannot accurately calculate wireless propagation characteristics in an underground mine when both transceiver antennas are near a tunnel wall simultaneously. So, we use hybrid RT method’s simulation value from reference^14^ and corresponding position’s measured value to compare with RT-FDTD method’s calculation accuracy near a tunnel wall simultaneously. We place a transmitting point near wall L with coordinate (− 2.36,0,0). Surface excitation source ’s distance from wall L is 1.2 m; and its distance from transmitting point along *z*-axis is 4 m. Both surface excitation sources have identical *y*-coordinates that are 1/4 of a tunnel’s height. We place each receiving point at a distance of 0.04 m from a tunnel wall along *z*-axis to simulate wireless propagation characteristics between two wireless communication base station antennas installed on same side of a tunnel wall.

The 700MHz frequency band is a traditional broadcasting and television system frequency band in China. As a golden frequency band for conducting mobile communication services, the 700MHz frequency band has good propagation characteristics and this band was adjusted for 5G communication by the Ministry of Industry and Information Technology of China in 2020. So we conducted many measurement tests underground at Shuangma Coal Mine of Ningxia Coal Industry CO., LTD; and measured wireless propagation characteristics for electromagnetic waves with frequencies of 580 MHz and 740 MHz when we placed transmitting and receiving antennas at different positions on a tunnel section to promote 5G communication system in underground mine.

**Figure 8:**
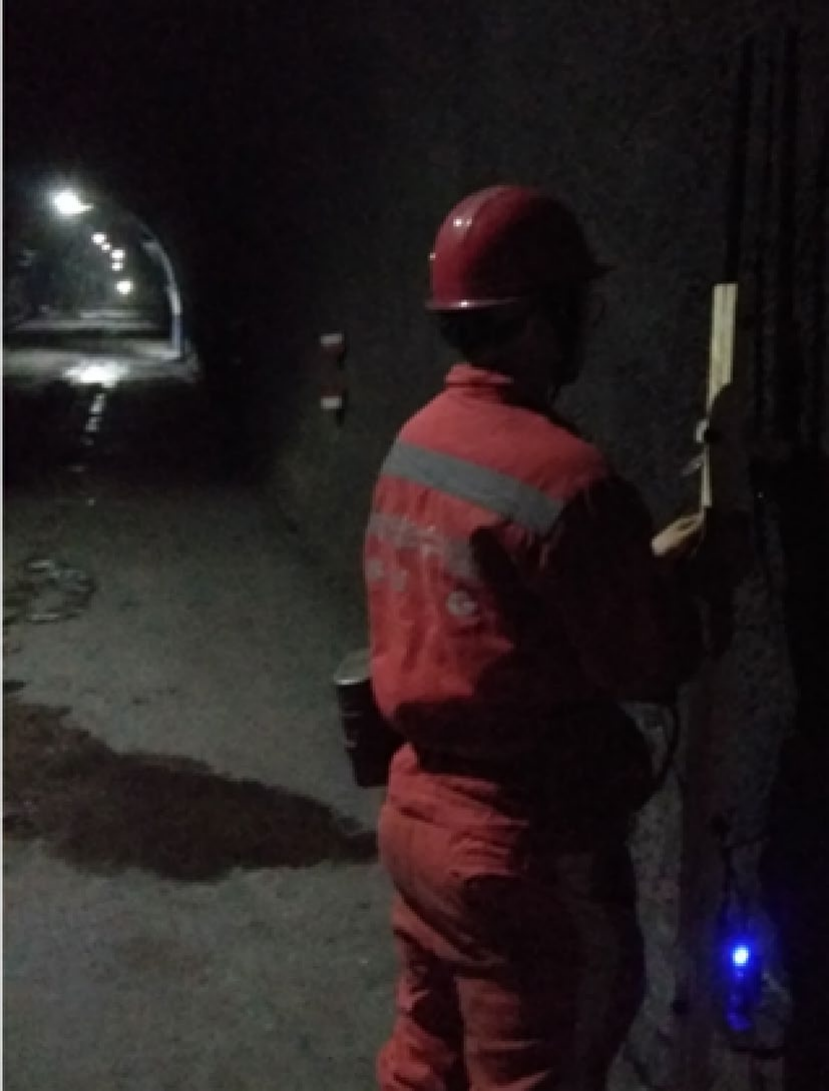
Measurement environment.

**Figure 9:**
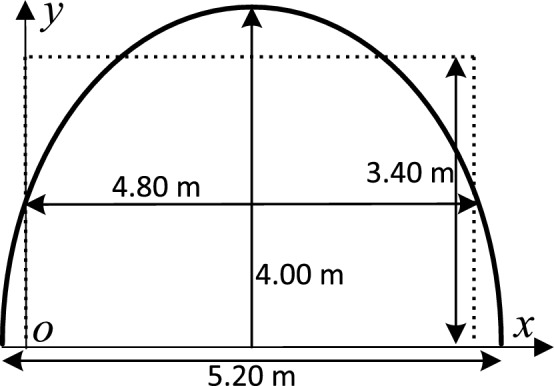
The section size of actual tunnel and equivalent rectangular tunnel.

Testing tunnel’s cross-section is horseshoe-shaped;and based on the research results in the reference^29^, we can equate it to a rectangular tunnel with width of 4.8 m and height of 3.4 m using area equivalence principle. Figures 8 and 9 show measurement environment and equivalent tunnel respectively. Measuring device’s RF transmitting power is 30 dBm; and receiving device’s sensitivity is − 92 dBm at a communication rate of 1 Mbps. We used a vertically polarized omnidirectional dipole antenna with characteristic impedance of 50 $\Omega$ and gain of 1 dBi. The distance from the transmitting and receiving antennas to the floor is 1.70 m and the max distance from the transmitting antenna to the receiving antenna is 200 m along the length direction of the tunnel.

**Figure 10:**
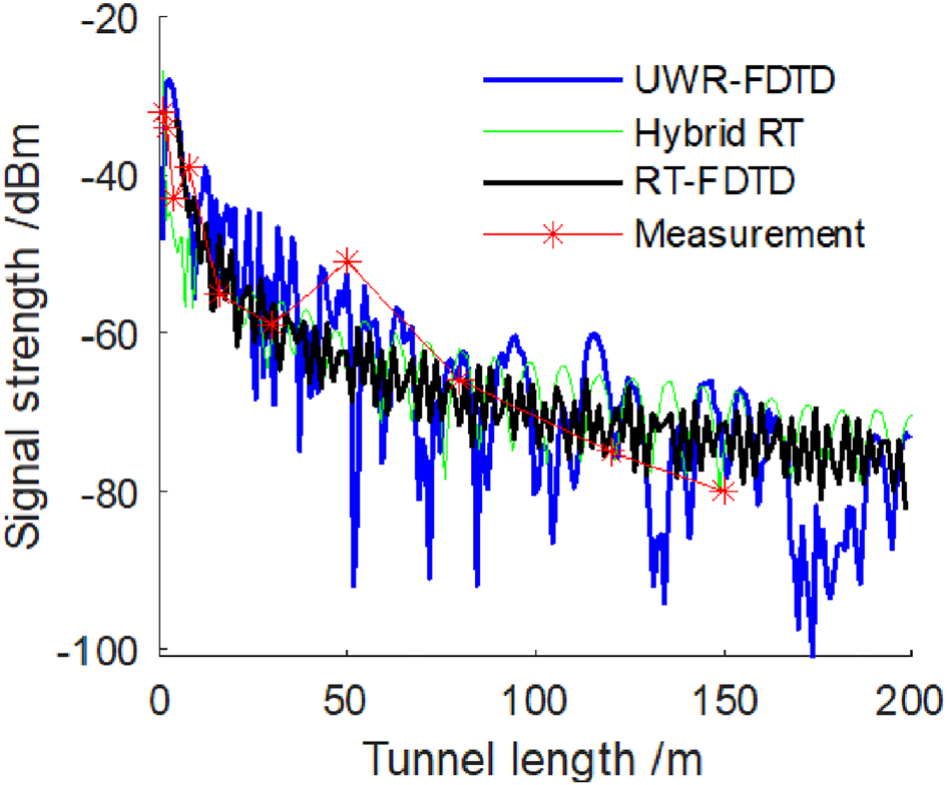
The signal strength of 580 MHz electromagnetic wave.

**Figure 11:**
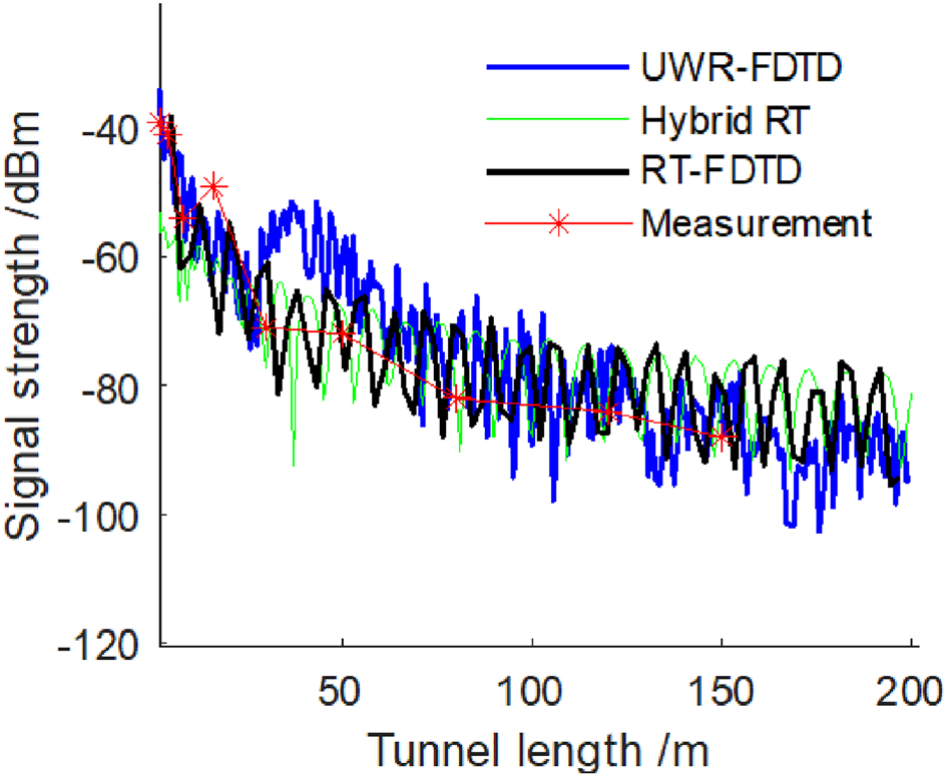
The signal strength of 740 MHz electromagnetic wave.

We place transceiver antenna at a distance of 0.04 m from same side’s tunnel wall. We compare simulation values calculated by UWR-FDTD method, mixed RT method and our RT-FDTD method with measured values. Figure 10 shows received signal strength for a 580 MHz frequency electromagnetic wave along length direction of a tunnel; and Fig. 11 shows received signal strength for a 740 MHz frequency electromagnetic wave.

Figure 10 shows that when both transceiver antenna and tunnel wall L are at a distance of 0.04 m from each other, three methods—UWR-FDTD, hybrid RT and our RT-FDTD calculate a consistent attenuation trend for 580 MHz electromagnetic wave signal strength along a tunnel’s length; and measurement results agree with this trend. Figure 11 shows similar results for 740 MHz electromagnetic wave’s received signal strength calculated along a tunnel’s length. Analysis results show that we can use our RT-FDTD method to calculate electromagnetic wave propagation characteristics when both transceiver antennas are near a tunnel wall simultaneously; and this solves large error problem in RT method’s simulation results for this scenario.

### Computational efficiency

We study computational efficiency of RT-FDTD, FDTD, UWR-FDTD methods for the same simulation tunnel through theoretical analysis and simulation experiments.

An electromagnetic wave’s wavelength is $\lambda$; and we discretize each wavelength into $N_d$ grids with each grid’s length being $N_d/\lambda$. For a 3D space with length L, width W and height H; we divide it into $N_{Yee}$ grids.

18 $$\begin{aligned} N_{Yee} =\left( \frac{N_{d} }{\lambda }^{3} \right) LWH \end{aligned}$$

We need to allocate memory space for each electromagnetic field component in a grid. An electromagnetic wave’s wavelength is fixed for a same frequency. To prevent numerical dispersion for an electromagnetic wave, we usually set the value of $N_d$ to be equal or greater than 10. Once we fix the value of $N_d$, we consider it as a constant. In this case, space size to be solved determine number of Yee grids. We use RT method in our RT-FDTD method to calculate finite direct and reflection paths between transmitting point and receiving point in a tunnel’s central area. We do not need to divide entire computing space into Yee grids or allocate memory space for electric and magnetic field components in these grids anymore. So theoretically our RT-FDTD method requires much less memory space than FDTD method.

We can set the distance from the surface excitation source $S_{RF1}$ to the tunnel wall to be 1/4 of a tunnel’s width, and we can calculate surface excitation source SRF1 by RT method. We use surface excitation source $S_{RF2}$’s z-directional coordinate position as a reference electric field strength; its value should not be too large—an integer between 1 and 4 is desirable. We consider surface excitation sources $S_{RF3}$’s and $S_{RF4}$’s sizes comprehensively based on required calculation for transceiver antenna’s position from a tunnel wall and computer memory’s size. Least ideal condition is when surface excitation sources and coincide with roof and floor respectively. In this case, our RT-FDTD method saves at least 1/4 memory space compared to traditional FDTD method. We can also reduce memory space required by this method by adjusting surface excitation sources $S_{RF3}$’s and $S_{RF4}$’s heights.

We calculate memory space and simulation time required by FDTD, UWR-FDTD and our RT-FDTD methods on same server for five electromagnetic wave frequencies: 580 MHz, 740 MHz, 900 MHz, 2100 MHz and 2600 MHz to calculate computing efficiency of these methods. Server CPU model is Intel(R) Core (TM) i7-7700K @ 4.20 GHz with 32 GB RAM. Simulated tunnel has length of 200 m, width of 4.8 m and height of 3.4 m; and spatial steps for Yee grid along *x*-axis, *y*-axis and *z*-axis are all 1/10 of an electromagnetic wave’s wavelength.

We set unit window length to be 1/8 of a tunnel’s length when we simulate three frequency electromagnetic waves—580 MHz, 740 MHz and 900 MHz—using UWR-FDTD method; this is best division window for UWR-FDTD method and most efficient calculation. If we divide unit window by highest efficiency for UWR-FDTD method when we simulate 2100 MHz and 2600 MHz frequency electromagnetic waves, required memory space exceeds 32 GB and we cannot calculate. We set unit window length to be 1/30 of a tunnel’s length in simulation experiment; and we trade simulation time for storage space to calculate 2100 MHz frequency electromagnetic wave using UWR-FDTD method.

We set distance from face excitation source to tunnel wall to be 1/4 of a tunnel’s width; distance from face excitation source to emission point to be 4 m; and height of face excitation source and tunnel to be 1/4 of a tunnel’s height when we use our RT-FDTD method to calculate above five frequency electromagnetic waves.

Table 1 shows required memory space and simulation time for those three methods. We estimate required memory space for FDTD method’s calculation of 2100 MHz and 2600 MHz frequency electromagnetic waves from a line fit to required memory space for FDTD method’s calculation of 580 MHz, 740 MHz and 900 MHz frequency electromagnetic waves in Table 1. Required memory space and simulation time for FDTD method’s calculation of these three frequency electromagnetic waves are what we need to run MATLAB programs.

**Table 1:** Memory space and simulation time required of three methods.

Frequency/MHz	Wavelength/m	Memory space/MB			Simulation time/h		
		FDTD	UWR-FDTD	RT-FDTD	FDTD	UWR-FDTD	RT-FDTD
580	0.52	4600	2400	4300	0.6	0.2	0.1
740	0.41	6000	2900	4600	1.5	0.3	0.1
900	0.33	9100	5200	5100	4.4	0.7	0.2
2 100	0.14	72,400	9600	5600	–	15.8	0.76
2 600	0.11	125,400	–	6100	–	–	1.6

As can be seen from the data in Table 1, compared with the traditional FDTD method, the performance of UWR-FDTD and RT-FDTD methods has been improved in terms of memory space and simulation time. However, UWR-FDTD method still requires a huge memory space to calculate higher frequency electromagnetic waves. It cannot calculate and simulate the propagation characteristics of 2600 MHz electromagnetic wave in the tunnel. We calculate the memory size of 580 MHz, 740 MHz and 900 MHz by FDTD method, and then we do the linear fitting of these memory values. According to the fitting data, the memory space required by FDTD method to calculate 2100 MHz and 2600 MHz electromagnetic wave is 72,400 MB and 125,400 MB, respectively. Because the computing space required to divide Yee grid is greatly reduced, the memory space required by RT-FDTD method to compute 2600 MHz electromagnetic wave is increased by 1800 MB compared with that required by 580 MHz electromagnetic wave, which is about 3 times. The simulation time is 16 times longer than that of 580 MHz electromagnetic wave. Under the same conditions, the memory space required by FDTD method to calculate 2600 MHz electromagnetic wave is 27 times that of 580 MHz electromagnetic wave. Because UWR-FDTD method only divides the unit window along the tunnel axis, but does not divide the tunnel section, the memory space required by the tunnel section alone exceeds the available memory space of the server when calculating the electromagnetic wave propagation characteristics of 2600 MHz. After we adopt the strategy of increasing simulation time in exchange for memory space, the simulation time required by the UWR-FDTD method to calculate this frequency is 23 times that of 900 MHz. We can complete the simulation calculation of 2600 MHz electromagnetic wave within 1.6 h using RT-FDTD method.

## Conclusions

1. The RT-FDTD method effectively combines the high efficiency of the RT method and the precision of the FDTD method, guided by the law of conservation of energy. This approach addresses the limitation of the RT method, which is not suitable when transmitting and receiving antennas are located on the same side of the tunnel. Moreover, it significantly reduces the demand for external resources required by the FDTD method, thereby improving the computational efficiency compared to using the FDTD method alone. This innovative method offers a promising solution for enhancing wireless communication in complex environments such as underground mine.
  The RT-FDTD method combines RT method’s high efficiency and FDTD method’s precision using law of conservation of energy. It solved the problem that the RT method is not suitable for transmitting and receiving antennas located on the same side of the tunnel at the same time. At the same time, it greatly reduced the demand for external resources of the FDTD method and improved computational efficiency of using the FDTD method alone.
2. The RT-FDTD method consistently calculates the signal strength of different frequency electromagnetic waves in line with results obtained from both the FDTD and UWR-FDTD methods, as well as measured values presented in this paper. This consistency holds true regardless of the location of the transmitting and receiving antennas. Given that communication equipment antennas are typically installed on the tunnel wall or roof, the RT-FDTD method proves particularly suitable for planning antenna positions of communication base stations in tunnels. This makes it a valuable tool for optimizing wireless communication in such complex environments.
3. The propagation characteristics of a 740 MHz electromagnetic wave were calculated using the FDTD, UWR-FDTD, and RT-FDTD methods in a simulated tunnel. The RT-FDTD method proved to be significantly more efficient, requiring only 1/15 of the simulation time needed by the FDTD method and one-third of the time required by the UWR-FDTD method. For 900 MHz electromagnetic wave, the RT-FDTD method costs only 1/22 of the simulation time needed by the FDTD method and one-third of the time required by the UWR-FDTD method. Due to the high memory demands of the FDTD and UWR-FDTD methods, these methods could not be used to calculate the propagation characteristics for a 2600 MHz electromagnetic wave. However, the RT-FDTD method was able to perform the same simulation in approximately 1.6 hours. These results highlight the efficiency and practicality of the RT-FDTD method in simulating wireless propagation characteristics in underground mine.

## Data Availability

Due to the support of our manuscript from the National Key R &D Program and the Henan Province Science and Technology Research and Development Program, the content and data are important components of both programs, and some data disclosure requires authorization and permission from the competent department. The dataset generated and/or analyzed during the current research period is not publicly available, but can be obtained from the corresponding author upon reasonable request.
