# Author Correction: A RT-FDTD method of analyzing wireless propagation characteristics in underground mine

**DOI:** 10.1038/s41598-024-66720-8

**Published:** 2024-07-08

**Authors:** Xiaoyan Song, Gaomin Zhang, Chang Zhou

**Affiliations:** 1 College of Electrical and Mechanical Engineering, Pingdingshan University, Pingdingshan, 467000 China; 2 College of Information Engineering, Pingdingshan University, Pingdingshan, 467000 China; 3 Information Technology Research Institute, China University of Mining and Technology-Beijing, Beijing, 100083 China; 4 School of Computer Science and Technology, Henan Open University, Zhengzhou, 450008 China

Correction to: *Scientific Reports* 10.1038/s41598-024-60151-1, published online 29 April 2024

In the original version of this Article Xiaoyan Song was incorrectly affiliated with ‘College of Information Engineering, Pingdingshan University, Pingdingshan, 467000, China.’ Their correct affiliation is listed below.

College of Electrical and Mechanical Engineering, Pingdingshan University, Pingdingshan, 467000, China.

The original Article has been corrected.

